# Rapid Heterotrophic Ossification with Cryopreserved Poly(ethylene glycol-) Microencapsulated BMP2-Expressing MSCs

**DOI:** 10.1155/2012/861794

**Published:** 2012-02-07

**Authors:** Jennifer Mumaw, Erin T. Jordan, Corinne Sonnet, Ronke M. Olabisi, Elizabeth A. Olmsted-Davis, Alan R. Davis, John F. Peroni, Jennifer L. West, Franklin West, Yangqing Lu, Steven L. Stice

**Affiliations:** ^1^Department of Animal and Dairy Science, Regenerative Bioscience Center, University of Georgia, 425 River Road, Athens, GA 30602, USA; ^2^Center for Cell & Gene Therapy, Baylor College of Medicine, One Baylor Plaza, Room N1010, Houston, TX 77030, USA; ^3^Department of Molecular Medicine, City of Hope Beckman Research Institute, 1500 East Duarte Road, Duarte, CA 91010, USA; ^4^Department of Large Animal Medicine, College of Veterinary Medicine, University of Georgia H-322, 501 D.W. Brooks Drive, Athens, GA 30602-7385, USA; ^5^Department of Bioengineering, Rice University, 6100 Main Street, Houston, TX 77005-1892, USA; ^6^State Key Laboratory for Conservation and Utilization of Subtropical Agro-bioresources, Animal Reproduction Institute, Guangxi University, Guangxi, Nanning 530004, China

## Abstract

Autologous bone grafting is the most effective treatment for long-bone nonunions, but it poses considerable risks to donors, necessitating the development of alternative therapeutics. Poly(ethylene glycol) (PEG) microencapsulation and BMP2 transgene delivery are being developed together to induce rapid bone formation. However, methods to make these treatments available for clinical applications are presently lacking. In this study we used mesenchymal stem cells (MSCs) due to their ease of harvest, replication potential, and immunomodulatory capabilities. MSCs were from sheep and pig due to their appeal as large animal models for bone nonunion. We demonstrated that cryopreservation of these microencapsulated MSCs did not affect their cell viability, adenoviral BMP2 production, or ability to initiate bone formation. Additionally, microspheres showed no appreciable damage from cryopreservation when examined with light and electron microscopy. These results validate the use of cryopreservation in preserving the viability and functionality of PEG-encapsulated BMP2-transduced MSCs.

## 1. Introduction

Bone is the second most transplanted tissue behind blood transfusions [1] with 500,000 people in the US and 2.2 million people worldwide receiving bone grafts per year [2]. Autologous bone grafting is currently considered the gold standard for treating nonhealing fractures [3], but multiple features make it less than ideal for long bone nonunion treatment. The most promising graft donor site, the iliac crest, is available in limited quantities [4]. Since long bone nonunions can require up to 30 mLs of marrow, the amount harvested from the iliac crest can be insufficient [5]. Bone grafting presents considerable risks to patients by increased surgical times and blood loss [6], with 1/3 of patients experiencing chronic pain 24 months after transplant [7], and recipients are at increased risk for donor site instability and fractures [8]. Additionally, large bone defects, like those received by soldiers injured in combat [9, 10], often do not heal without surgical intervention and can end in an undesirable outcome such as amputation [11].

Bone morphogenetic protein 2 (BMP2) is a potential therapeutic that can fill the need for bone healing. Recombinant BMP2 can induce rapid ossification in orthopedic applications [12, 13] but has a relatively short half-life, must be administered at high dosages, and continually maintained to promote extensive and expedited bone regeneration [14–16]. Having a fast and maintained release/production of BMP2 as an off the shelf therapeutic might be used without the morbidity associated with bone grafting, reduce recovery time, and minimize the need for future surgeries. Mesenchymal stem cells (MSCs) could be a vector for delivering BMP2; MSCs have several potential advantages: they can be easily harvested from adult bone marrow [17] and adipose tissue [18, 19], are immunomodulatory [20–22], have allogeneic tolerability [21], are easily expanded *in vitro*, and can differentiate into bone even after long-term culture [19, 23].

Cellular encapsulation with genetically engineered cells producing BMP2 in a PEG hydrogel for bone regeneration was developed to extend expression of BMP2 *in vivo* [24]. PEG is an attractive material for biomedical applications with biocompatibility in multiple tissues [25–29]. Additionally, the mechanical properties of PEG can be altered to replicate that of soft tissue [30, 31]. As soft tissue injury often occurs at the same time as long-bone injury, healing involves the regeneration of both tissues [32]. Mimicking *in vivo* soft tissue has been shown to be more permissive for physiological healing in creating an environment permissive for angiogenesis [33], a vital component for correct bone healing [34]. PEG also has the ability to be made biodegradable in tissues through manipulations of the peptide sequences linking PEG moieties [35–38] or incorporation of extracellular matrices [39] which makes the structure cleavable through proteolytic processes, allowing the polymer and encapsulated cells to be removed by the body during the healing process. Initial studies with BMP2-transduced cells microencapsulated in PEG hydrogels have been shown to be superior to unencapsulated cells through the extended presence of BMP2-producing cells at the site of treatment and increased induction of heterotopic ossification (HO) in the mouse [40]. This therapy has applications in human medicine for replacing or supplementing current technologies for increasing the rate of bone healing; however, processes to make the PEG hydrogel-microencapsulated cells available for immediate use are lacking.

Cryopreserved cells can be stored as “ready to use” prior to the therapeutic application [41], and cryopreservation of cells microencapsulated in PEG hydrogels would also enhance and widen their therapeutic uses because testing of preparations could be conducted well in advance in controlled good manufacturing practices (GMP) facilities for distribution to the clinical setting. Cryopreservation of cells microencapsulated in alginate has previously shown success in maintaining cell viability, stem cell characteristics, and cellular recovery [42–45]. Cryopreservation allows for thorough testing of the encapsulated MSCs with the ability to thaw samples for validating cell viability, therapeutic protein concentration, sterility, and microbead integrity for ensuring that the highest-quality production has been performed. However, the post thaw effects of PEG hydrogel microencapsulation on cell survival, transgene expression, microbead integrity, and biological activity have not been previously investigated.

Pigs and sheep are suitable models for human bone studies with long-bone dimensions [46, 47] and structure [48, 49] that are similar to humans. Pigs have been shown to have similarities in bone remodeling [48] while sheep provide a comparable model for bone in growth into osteoconductive biomaterials [50]. Using MSCs isolated from both pigs and sheep we have explored the possibility of cryopreserving PEG hydrogel-microencapsulated MSCs expressing BMP2. The cryopreservation of the cells within the microspheres showed no reduction in viability in comparison to nonpreserved microencapsulated MSCs, and the encapsulated cellular spheres showed no physical damage resulting from cryopreservation. It was also found that cell lines from various donors may have different potentials in genetic modification and transgene production. Using this process genetically modified cryopreserved MSCs producing BMP2 maintained function as seen through initiation of bone formation in an *in vivo* model for HO. While optimization for increasing cell viability in PEG is still required, these results demonstrate that PEG hydrogel microspheres have the potential to be manufactured for “off-the-shelf” therapeutic use.

## 2. Material and Methods

### 2.1. MSC Isolation and Culture

Porcine MSCs were isolated previously [51], and ovine MSCs were isolated with the same plate adherency techniques from healthy female ewes as previously described. Briefly, MSCs were isolated from bone marrow aspirates with 0.25 mLs acid citrate dextrose per mL of bone marrow. MSCs were plated by mixing in a 3/5 ratio with MSC culture medium: Alpha-Minimum Essential Medium (Gibco), 10% defined fetal bovine serum (Hyclone), 2 mM L-glutamine, 50 U/mL penicillin (Pen), 50 *μ*g/mL streptomycin (Strep; all from Gibco/Invitrogen), and plating on tissue culture flasks. Cultures were maintained at 37°C and at 5% CO_2_. MSCs were harvested using 0.05% trypsin (Gibco) and replated at 5,000 cells/cm^2^ upon reaching 80–90% confluency (60,000–75,000 cells/cm^2^).

### 2.2. Lineage Differentiation

Differentiation was performed using previously established protocols [51] with moderate alterations: for adipogenic and osteogenic differentiation, 36,000 cells/cm^2^ were plated in 6-well plates. MSCs were allowed to reach confluency and then switched to adipogenic or osteogenic medium: adipogenic differentiation was initiated in induction medium: Dulbecco's Modified Eagle Medium (DMEM) high glucose (Hyclone), Pen/Strep, 1 *μ*M dexamethasone, 10 *μ*g/mL insulin, 200 *μ*M indomethacin, 500 *μ*M 3-isobutyl-1-methyl-xanthine (Sigma), and 10% FBS (Hyclone) for 3 days followed by 14 days in differentiation medium: DMEM high glucose, Pen/Strep, 10 *μ*g/mL insulin, and 10% FBS. Differentiated plates were stained with 0.7% Oil Red O. Osteogenic differentiation was performed using HyClone Advance STEM Osteogenic Differentiation kit (Thermo Scientific) with medium changes every third day for 21 days, and samples were stained with Von Kossa. For chondrogenic differentiation potential validation of MSCs, 3 × 10^6^ cells were pelleted in 15 mL conical tubes and then changed to chondrogenic medium DMEM (high glucose), 100 nM dexamethasone, Pen/Strep, 50 *μ*g/mL ascorbic acid, 40 *μ*g/mL L-proline, 1 × ITS + 1 supplement (recombinant human insulin, human transferring, sodium selenite, bovine serum albumin, and linoleic acid), 1 mM sodium pyruvate (all from Sigma), and 10 ng/mL TGF-*β*3 (R&D Systems). Medium was changed every third day for 14 days. Micromasses were stained with Alcian Blue.

### 2.3. Proliferation

Proliferation was determined using manual cell counts with 0.04% trypan blue (Sigma) live/dead exclusion staining, and only live MSCs were counted. MSCs were plated at 6,000 cells/cm^2^ and harvested for counts 12 hours following plating. This initial count was deemed time 0, and MSCs were harvested and counted at 12, 24, 36, 48, and 60 hours after the initial count. All counts were performed in triplicate.

### 2.4. Microencapsulation

Using techniques previously described [40, 52], MSCs were harvested using 0.05% trypsin and counted on a hemocytometer using 0.04% Trypan Blue (Sigma) staining for live/dead exclusion. 3.5 × 10^4^ MSCs/*μ*L were suspended in an aqueous hydrogel solution containing 0.1 g/mL 10 kDa PEG diacrylate (PEGDA), 1.5% (v/v) triethanolamine/HEPES-buffered saline, 37 mM 1-vinyl-2-pyrrolidinone, 0.1 mM eosin y, and 9 mM pluronic acid. For photo initiation, 1.17 M 2,2-dimethoxy-2-phenyl acetophenone was dissolved in 1-vinyl-2-pyrrolidinone, and 3 *μ*L of this solution was added per mL of sterile mineral oil (Sigma-Aldrich). Hydrogel/cell suspension was mixed with mineral oil containing the photoinitiator and vortexed for 2 seconds while being exposed to white light from a Metal Halide Illuminator (Edmonds Optics) followed by another 18-second exposure with mild mixing. Microencapsulated MSCs were separated from the oil with four washes in MSC culture medium with 5-minute centrifugation at 1350 RPM and decanting between washes.

### 2.5. Viability Assays

Cell viability was assessed using the LIVE/DEAD Viability/Cytotoxicity Kit for Mammalian MSCs (Invitrogen). Images were taken using TCS SP5 Spectral Confocal Microscope (Leica). 3 sets of images were taken per condition with 30 images in each set with an average of 87,210 cells being counted in each treatment using Image J (NIH).

### 2.6. Cryopreservation and Thawing

MSCs and microspheres were frozen in MSC culture medium containing 10% DMSO. The MSCs were frozen in controlled rate freezing containers, Mr. Frostys (Nalgene labware) for 4–24 hours at −80°C and then transferred to liquid nitrogen. Vials were thawed in a 37°C water bath with constant swirling. The MSCs were resuspended with medium immediately following loss of ice from cell/microsphere suspension. To limit confounding factors microspheres were thawed using a ratio of twenty percent physical cell loss. This number was established on the percentage of cells lost during cryopreservation and thawing processes.

### 2.7. Adenoviral Transduction Optimization and BMP2 Quantification

First-generation human type 5 adenoviruses containing the E1–E3 deletion were constructed with human cDNA for BMP2 inserted in the E1 region. MSCs were harvested and plated one day prior to transductions. Transductions were performed as described previously [51] with minor changes. Upon reaching a density of 36,000 cells/cm^2^ the MSCs were prepared for transduction. To increase cell-viral interactions transductions were performed in reduced medium volumes. Medium was changed with replacement of 32% of normal culture volume of MSC culture medium. Transduction medium was made equaling 20% of normal culture volume with Alpha MEM medium with 2 mM L-glutamine and mixed with 0.72% Genejammer (Agilent Technologies) and allowed to incubate for 5 minutes at room temperature. The virus was then added to the transduction medium and allowed to incubate for 10 minutes at room temperature. For optimization of BMP2 transduction, transductions were performed using 5,000, 7,500, 10,000, and 15,000 vp/cell (See Supplementary Figure  1 in Supplementary Material available online at doi: 10.1155/2012/861794). The remaining experiments were performed with 15,000 vp/cell. The transduction mixture was then added to the cell culture dropwise around the plate. After four hours the culture volume was brought up to normal volume with MSC culture medium. MSCs were harvested 24 hours after the transduction. The MSCs were replated at 36,000 cells/cm^2^ or microencapsulated then replated at 36,000 cells/cm^2^. BMP2 was quantified from harvested medium using a BMP2 ELISA (R&D systems).

### 2.8. Scanning Electron Microscopy and Light Microscopy

Both freshly prepared and cryopreserved microspheres containing ovine MSCs were immersion fixed using 2.5% glutaraldehyde in PBS for one hour. The MSCs were washed three times with PBS and postfixed in 1% osmium tetroxide diluted in 5% sucrose and PBS for 45 minutes. The microspheres were washed three times with distilled water and then carried through an alcohol dehydration series. The MSCs were critically point-dried using a Samdri model 780-A (Tousimis). A 153 Å thick coating of gold was placed on the samples using SPI Module Sputter Coater (Structure Probe). The images were taken on 1450EP environmental Scanning Electron Microscope (Carl Zeiss).

### 2.9. Heterotopic Bone Assay

All animal studies were performed with Baylor College of Medicine Institutional Animal Care and Use Committee approval. Female nonobese diabetic/severely compromised immunodeficient mice (NOD/SCID; 8–12 weeks old; Charles River Laboratories) were injected with 3 × 10^6^ microencapsulated MSCs either freshly prepared or cryopreserved and thawed from ovine B MSCs. Microspheres were injected into the hind-limb quadriceps of 4 mice per group (*n* = 8). Animals were euthanized at 2 weeks and X-rayed. The tissue was then harvested and fixed in formalin.

### 2.10. Microcomputed Tomography

Microcomputed tomography (micro CT) was obtained from the legs injected with the microsphere preparations. The legs were examined at a 15 mm resolution (eXplore Locus SP; GE Healthcare, London, ON, Canada). A hydroxyapatite phantom was scanned alongside each specimen and was used to convert the scan data from arbitrary units to units of equivalent bone density. The three-dimensional region of interest was defined for each animal to separate HOs from the normal skeletal structures. The threshold for tissue within the region of interest was set to exclude any tissue with a density less than 100 mg/cc, and the volume of tissue was calculated as a total amount of mineralized tissue.

### 2.11. Graphical Representation and Statistics

Graphs were made in Prism (Graphpad), and all statistics were also done in Prism. Statistics comparing BMP2 production were performed using 2-way ANOVA with Bonferonni posttest. Viability comparisons were done with 1-way ANOVA using Tukeys posttest. Doubling times were calculated using the exponential growth equation in Prism, and comparison of doubling times was done with 1-way ANOVA with Bonferroni posttest. HO volumes were compared using a Student's *t*-test.

## 3. Results

**Figure 1:**
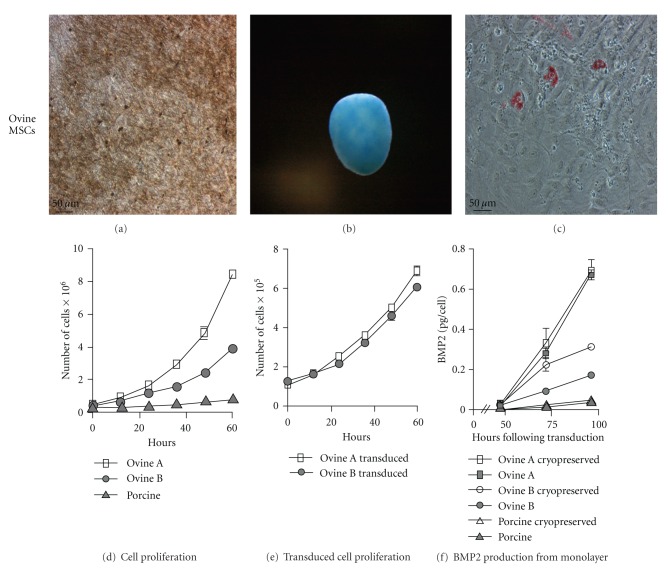
Characterization of ovine MSCs and proliferation and BMP2 transduction of ovine and porcine MSCs: ovine MSCs (a) osteogenic differentiation is seen through dark staining of calcium depositions by Von Kossa silver nitrate staining. (b) Chondrogenic differentiation with chondroitin sulfate proteoglycans stained blue with Alcian Blue staining, and (c) adipogenic differentiation as seen through intracellular lipid staining with Oil Red O. (d) Ovine A, ovine B, and porcine cell line proliferation rates. (e) Proliferation rates of ovine A and ovine B following transduction with 15,000 vp/cell of adenoviral BMP2. (f) Transduction with 15,000 vp/cell adenoviral BMP2 production from ovine and porcine MSCs both with and without cryopreservation.

Lineage differentiation of porcine MSCs used in this study was previously validated [51]. Ovine MSCs isolated through plate adherence from bone marrow aspirates were differentiated using previously developed protocols and were capable of osteogenic, chondrogenic, and adipogenic differentiation (Figures 1(a), 1(b), and 1(c)). Ovine MSCs underwent 21 days of osteogenic differentiation and showed evidence of calcium deposition as seen through Von Kossa silver nitrate staining (Figure 1(a)). After 14 days of chondrogenic differentiation the micromasses exhibited sulfate proteoglycans as seen through Alcian Blue staining (Figure 1(b)), indicating the presence of chondrocytes. At 17 days of adipogenic differentiation, lipid droplets were visible within MSCs through Oil Red O staining (Figure 1(c)), validating the capacity of these derived MSCs to differentiate into all three mesenchymal stem cell lineages.

During the expansion phase it was noted that the ovine A MSCs proliferated more rapidly than the ovine B MSCs and porcine MSCs. To examine the differences in proliferation rates of the cell lines, 5 counts at 12 hour intervals were used to determine proliferation rates. Ovine A MSCs had a doubling time of 15.2 (±0.7) hours (*R* ^2^ = 0.9813), ovine B MSCs had a doubling time of 19.7 (±1.5) hours (*R* ^2^ = 0.9867), and porcine MSCs had a doubling time of 34.5 (±3.2) hours (*R* ^2^ = 0.9755). The doubling times from each line were all statistically different (*P* < 0.05) (Figure 1(d)). To understand the effect transduction had on the proliferation rates, doubling times of ovine A and ovine B MSCs were determined by plating the MSCs 24 hours after transduction and counting as described for the nontransduced cells. Adenoviral BMP2-transduced ovine A and ovine B MSCs showed a significant reduction in the proliferation rates from the nontransduced MSCs (*P* < 0.05) (Figure 1(e)) with a doubling time of 24.1 (±2.1) hours (*R* ^2^ = 0.9827) and 25.4 (±2.2 hours) (*R* ^2^ = 0.9820), respectively.

To determine the ability of the MSC to produce BMP2 following adenoviral transduction and the effect of cryopreservation on BMP2 production, monolayers of MSCs were transduced with 15,000 viral particles/cell. 15,000 vp/cell was chosen based on the highest BMP2 production from optimization of 5,000, 7,500, 10,000, and 15,000 vp/cell (*P* < 0.05) (Supplementary Figure  1). The MSCs were replated 24 hours after transduction or cryopreserved. Medium was harvested from cultures every 24 hours for 72 hours and quantified for BMP2 expression (Figure 1(f)). The cell lines showed a significant donor effect (*P* < 0.001) with ovine A MSCs producing the most BMP2. Ovine B MSCs had a significant increase in BMP2 expression from cryopreserved samples at 48 and 72 hours (*P* < 0.001).

**Figure 2:**
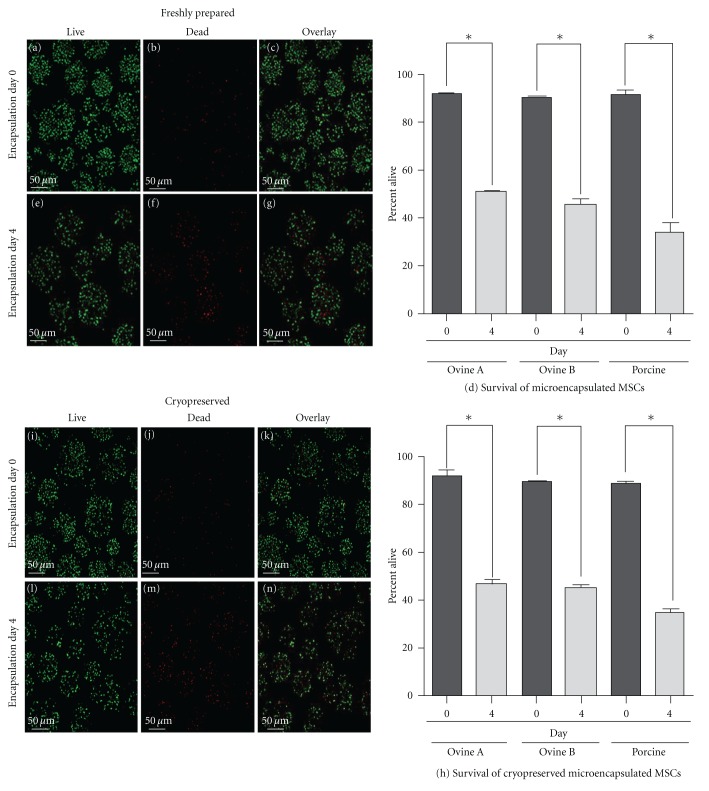
Viability of MSCs on day 0 and 4 after encapsulation with and without cryopreservation: MSCs were stained with calcein AM for live (green) and ethidium homodimer for dead (red). On the day of microencapsulation ovine A MSCs stained for (a) live, (b) dead, and (c) overlay. And on day 4 following microencapsulation (e) ovine A MSCs stained for live, (f) dead, and (g) overlay. (d) Graphical representation of counts of 90 images. Cryopreserved ovine A encapsulated MSCs on day of thaw (i) live, (j) dead, and (k) overlay. Day 4 postthaw ovine A MSCs stained for (l) live, (m) dead, and (n) overlay. (h) Graphical representation of counts of 90 images (averaging 87,000 cells per group). (*P < 0.0001).

**Figure 3:**
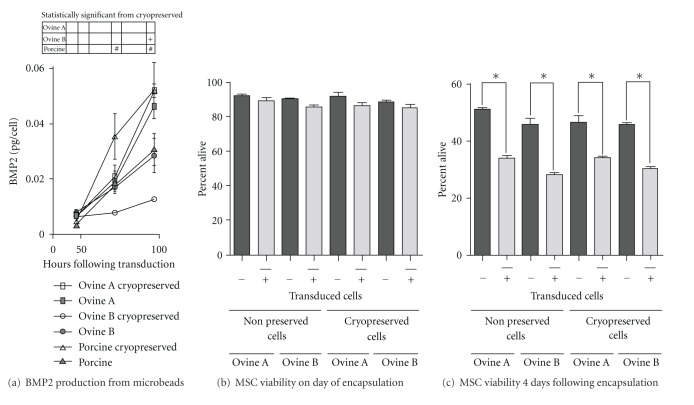
BMP2 production in microencapsulated MSCs. (a) Cells were transduced with 15,000 vp/cell adenoviral BMP2 prior to microencapsulation and plated out freshly or cryopreserved. (^+, #^ *P* < 0.01), (b) ovine A and ovine B cryopreservation and BMP2 transduction effect on MSC viability on day of microencapsulation (day of thaw for cryopreserved samples), and (c) 4 days after microencapsulation (*P* < 0.05).

To examine the effect of cryopreservation on the survival of microencapsulated MSCs, the viability of MSCs microencapsulated in PEG hydrogels was assessed using a live/dead assay which stains the cytoplasm of live MSCs with calcein AM (Figures 2(a), 2(e), 2(i), and 2(l)) and the dead MSCs DNA with Ethidium Homodimer (Figures 2(b), 2(f), 2(J), and 2(m)). No statistical difference was seen in the cell viability between the freshly prepared MSCs and the cryopreserved MSCs, but a significant reduction in cell viability was observed between day 0 and day 4 after-microencapsulation in both freshly prepared and cryopreserved microspheres (*P* < 0.0001) (Figures 2(d) and 2(h)). When microencapsulated, BMP2-transduced MSCs produced a reduced quantity of BMP2 (Figure 3(a)) when compared to monolayer BMP2-transduced MSCs (Figure 1(b)) at 72 and 96 hours after transduction (*P* < 0.05). Porcine microencapsulated BMP2-producing MSCs showed an increase in BMP2 production at 72 (*P* < 0.01) and 96 hours (*P* < 0.001) following transduction, and cryopreserved ovine B MSCs had a reduction in the quantity of BMP2 produced at 72 and 96 hours after transduction (*P* < 0.01) (Figure 3(a)). Ovine A MSCs had no difference between the cryopreserved and freshly prepared microencapsulated MSC BMP2; within the ovine lines, ovine A MSCs produced significantly more BMP2 than ovine B MSCs at 96 hours after transduction (*P* < 0.01) under both conditions (Figures 1(b) and 3(a)). BMP2 transduction has no effect on viability immediately following microencapsulation (Figure 3(b)), but the BMP2 microencapsulated MSCs did have reduced viability at day 4 (*P* < 0.05) when compared to the nonmodified MSCs (Figure 3(c)).

**Figure 4:**
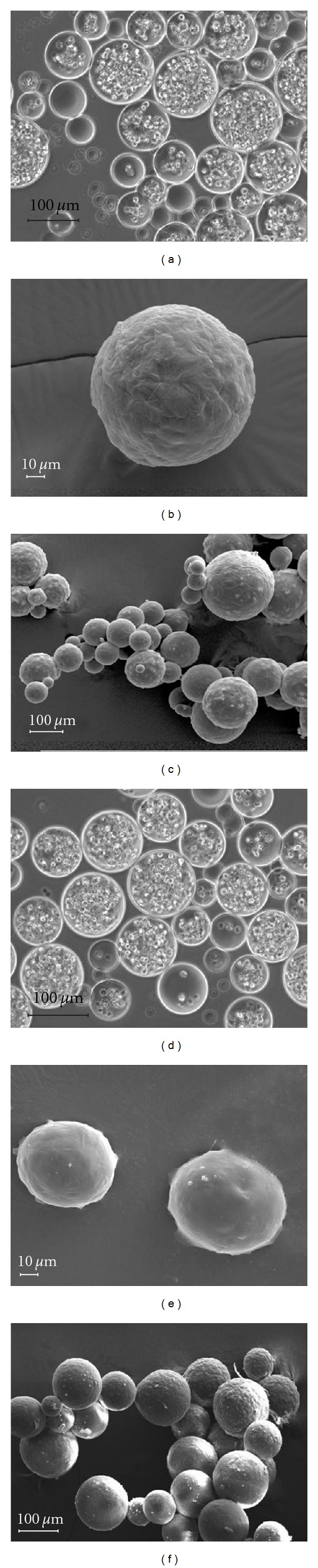
Structural analysis of freshly prepared and cryopreserved encapsulated MSCs: (a) phase contrast of microencapsulated ovine A MSCs showed clear borders on microbeads. (b) SEM of MSC microbeads showed a uniform surface. (c) SEM of MSC microbeads of all sizes showed uniform structure. (d) Phase contrast of cryopreserved microencapsulated MSCs did not show appreciable damage. (e) SEM of cryopreserved MSC microbeads showed a uniform surface. (f) SEM of cryopreserved microencapsulated MSCs showed no damage to beads of various sizes.

The integrity of the microspheres was examined following cryopreservation through scanning electron microscopy and light microscopy. The light microscopy images (Figures 4(a) and 4(d)) show the perimeter of the bead containing encapsulated ovine MSCs as being one contiguous surface with no rough edges. Additionally high-magnification images of the ovine microspheres demonstrated that the spheres possess uniform surfaces with no loss of integrity (Figures 4(b) and 4(e)). Cryopreservation did not result in any changes in the surface morphology of microencapsulated MSCs (Figures 4(c) and 4(f)).

**Figure 5:**
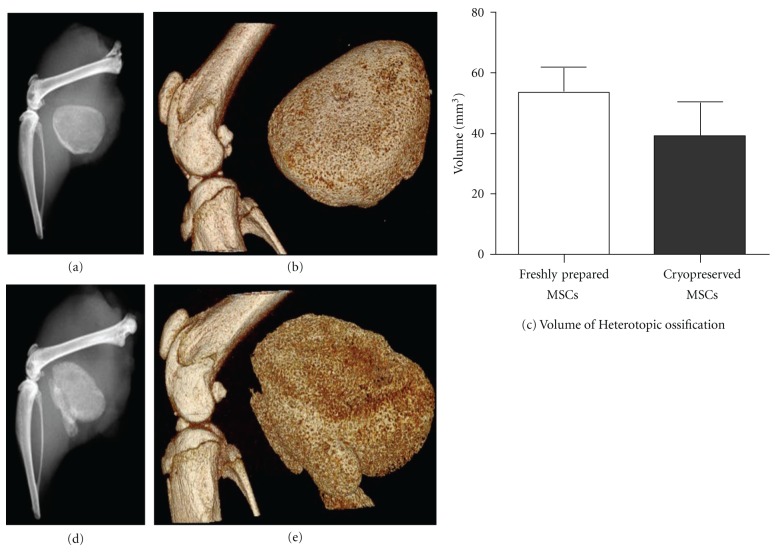
BMP2-transduced microencapsulated MSCs bone formation in a mouse model for heterotopic ossification. 3 × 10^6^ ovine B MSCs transduced with 15,000 vp/cell were injected into the hind limb of a NOD/SCID mouse. The resulting heterotopic ossification was observed by X-ray and MicroCT for (a) and (b) for freshly prepared BMP2 microencapsulated MSCs and (d) and (e) for cryopreserved BMP2 microencapsulated MSCs. (c) The volume of the resulting heterotopic ossification was not different between the two groups.

In the rodent model for HO, BMP2-transduced encapsulated microspheres are capable of producing bone as seen through radiographs two weeks following injection (Figures 5(a) and 5(d)). Additionally, volume rendering analysis through micro-CT of the HO produced by the freshly prepared microspheres (Figure 5(b)) and the cryopreserved microspheres (Figure 5(e)) did not exhibit differences in mineralized tissue volume (Figure 5(c); (*P* = 0.67)).

## 4. Discussion

A major hurdle in developing clinical treatments for bone injury is establishing methods to render the therapeutics widely applicable and readily available for clinical use. The recipient of a cell therapy, like organ transplantation, is at risk for graft rejection, and research has increased in cell microencapsulation as an immunoisolation technique in favor of immunosuppressants to modulate this immunological process [27, 53, 54]. 10 kDa PEGDA was chosen for this project based on previous data supporting its ability to allow proteins of 66,776 kDa molecular weight to pass through polymerized PEGDA, but still prevent the passage of IgG antibodies through the structure [55]. In this study we demonstrated for the first time that primary MSCs could successfully be cryopreserved in PEG hydrogel microspheres. This is a valuable progression in the movement of PEG hydrogel microencapsulation procedures from the bench top to the bedside. The combined microencapsulation and cryopreservation method yields high MSC viability after thaw, similar to alginate and sodium cellulose sulfate cell microencapsulation techniques [44, 56–58]. However, unlike previous reports of damage in alginate microcapsules during the cryopreservation process [11, 59, 60], PEG hydrogel microspheres did not show any appreciable damage upon removal from cryopreservation when examined by both light and scanning electron microscopy. Compromises in the integrity of the microsphere can result in exposure of the microencapsulated MSCs and initiation of an immune rejection [61]. The maintenance of integrity and cell viability seen with the cryopreserved microencapsualted MSCs is likely due to both the size and composition of the microbeads. Cryopreserved microencapsulated BMP2-transduced MSCs maintained their potential to form bone in a mouse model for HO, indicating that these preparations can be stored with no adverse effects on quality of the treatment. These features will eventually allow for production of a human-based product at GMP facilities with distribution to clinics. The viability of the primary MSCs was adversely affected by adenoviral genetic modification stressors in transduction and microencapsulation processes. For microencapsulated BMP2-transduced MSCs to be a viable treatment for long-bone injury extended BMP2 production will be required [16]. Here we show that the viability of the genetically modified microencapsulated MSCs was reduced to less than 40% by day 4 which may severely limit their therapeutic potential. Both inclusion of extracellular matrix proteins [62] and choosing cell lines with more substantial viability [40] are two potential methods that can be incorporated to increase cell viability and potentially increase the duration of BMP2 production. Additionally, the concentrations of Eosin Y, the photosensitizer, and triethanolamine, the initiator, may increase the loss of cell viability. These two compounds are vital for polymerization of the hydrogels, but at the concentrations used are toxic to MSCs [63].

The MSC line used for adenoviral BMP2 transduction can significantly impact the amount of BMP2 expressed. Since limited numbers of MSC lines between and within species were used here, a specific conclusion among individual MSC lines would be premature; however in general we found significant donor variation which affected both the rates of proliferation and BMP2 production from the MSC lines. This suggests that cell line selection may have an impact on the time required to expand cells in culture and the quantity of therapeutic BMP2 produced. Since the amount of BMP2 expression and rate of proliferation followed the same trend, a shorter cell cycle time may be an indicator of cell lines that are more amenable to higher rates of transduction. Since adenovirus is most effective at transducing cells in the S phase [64], cells with a shorter doubling time would be more likely to pass through S phase in the presence of active virus. There was less difference in BMP2 production between all lines following microencapsulation, but a difference between the ovine lines was still observed. This again indicates that MSC line-to-line variability significantly impacts the amount of BMP2 produced and that optimization of BMP2 expression may need to be conducted for each batch or lot of MSC collected regardless of prior validation.

The cryopreservation process did not affect the biological activity of the BMP2-transduced microencapsulated MSCs, and the volume of the microencapsulated BMP2-producing MSCs had similar quantities of induced heterotopic bone as seen previously with BMP2-producing microencapsulated fibroblast cells in NOD/SCID mice [40]. This study, like that of the fibroblast studies, used NOD/SCID mice to prevent interspecies rejection of the microencapsulated cells in releasing foreign proteins in the host. These results indicate that MSCs can be interchangeable with fibroblast cells in production of the therapeutic BMP2, and this flexibility can allow for the incorporation of immune modulatory characteristics of the MSCs in combination with transgene expression. Ultimately, cryopreservation of microencapsulated cells allows for greater application of derived therapeutics. This process facilitates the storage of sizable lots of characterized product for “off-the-shelf” regenerative cell therapies, ensuring the safest and most efficacious application of microencapsulated cellular therapy.

## 5. Conclusions

Microencapsulation of MSCs holds much promise for therapeutics in diseases without current effective treatments. To move these treatments forward, methods for preserving and long-term storage of microencapsulated MSCs to allow for “off-the-shelf” therapeutics are necessary. The cryopreservation of MSCs microencapsulated in PEG hydrogels did not reduce cell viability between the cryopreserved and freshly prepared MSCs, both with and without genetic modification, and did not demonstrate any physical damage resulting from the cryopreservation process. Cryopreservation does not induce any negative effects on the microencapsulated MSCs ability to form bone; however, the microencapsulated MSCs did have reduced viability following adenoviral transduction indicating a need for incorporating methods that increase viability of microencapsulated MSCs to prolong protein production. Donor-to-donor variability results in significant differences in transgene production, making cell line choice important for optimizing gene expression. This demonstrates that genetically engineered microencapsulated MSCs have potential for being used as a treatment method for clinical applications.

## Supplementary Material

Ovine MSCs were transduced with increasing concentrations of adenoviral BMP2. MSCs were transduced with 5,000, 10,000, and 15,000 vp/cell and the amount of BMP2 produced by the MSCs was quantified. The amount of BMP2 produced by the MSCs showed a linear trend with respect to viral particles transduced.
