# Supplementary figures and images for: Rapid Heterotrophic Ossification with Cryopreserved Poly(ethylene glycol-) Microencapsulated BMP2-Expressing MSCs
Source: Int J Biomater. 2012 Feb 7;2012:861794. doi: 10.1155/2012/861794 (PMC3296315; doi:10.1155/2012/861794)

## Slide 1
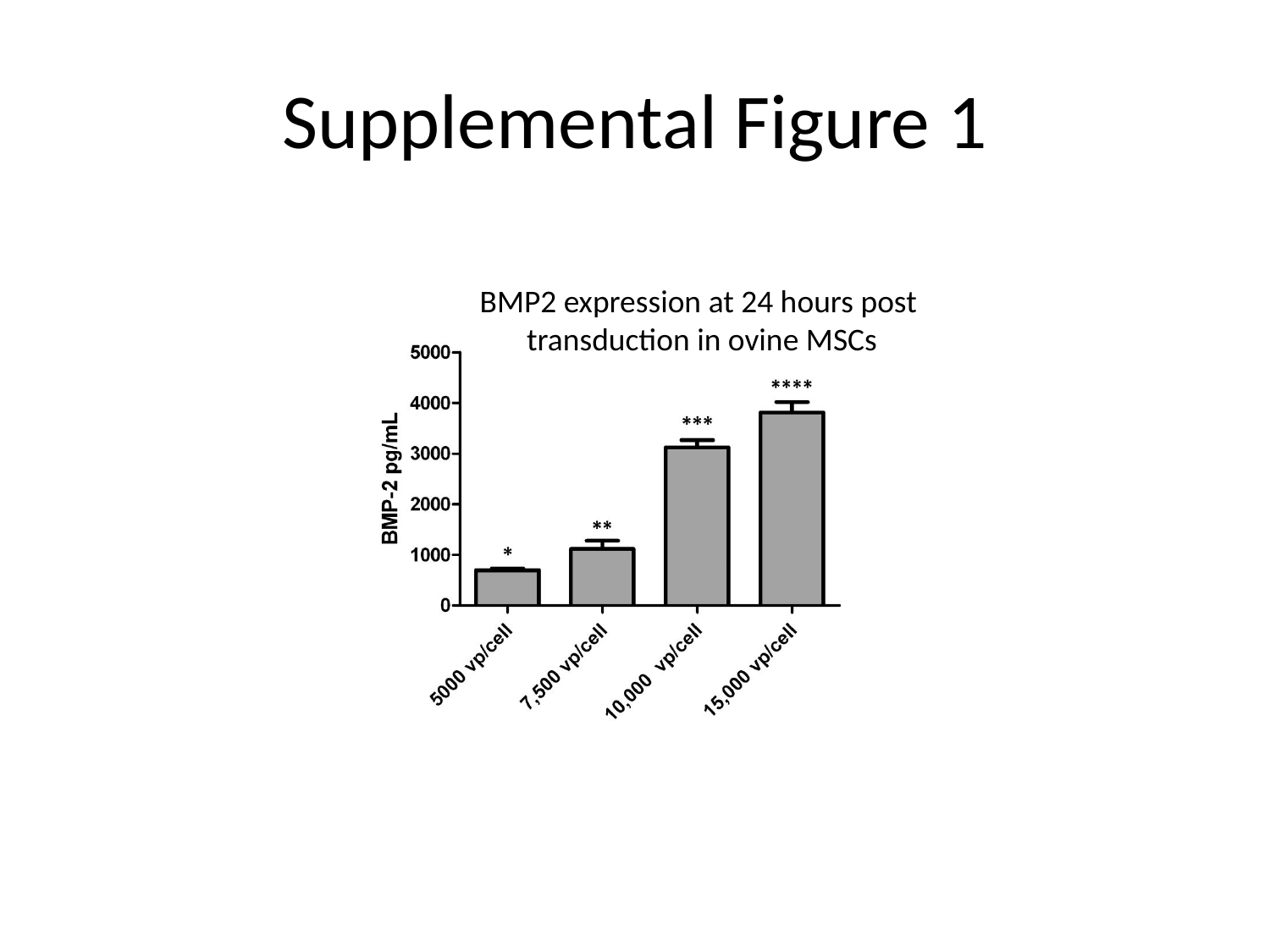

# Supplemental Figure 1
BMP2 expression at 24 hours post
 transduction in ovine MSCs

Supplement: Supplementary file 1 — Ovine MSCs were transduced with increasing concentrations of adenoviral BMP2. MSCs were transduced with 5,000, 10,000, and 15,000 vp/cell and the amount of BMP2 produced by the MSCs was quantified. The amount of BMP2 produced by the MSCs showed a linear trend with respect to viral particles transduced. [file 861794.f1.pptx]
